# Revisiting the Characterization of Resting Brain Dynamics with the Permutation Jensen–Shannon Distance

**DOI:** 10.3390/e26050432

**Published:** 2024-05-20

**Authors:** Luciano Zunino

**Affiliations:** 1 Centro de Investigaciones Ópticas (CONICET La Plata-CIC-UNLP), 1897 Gonnet, La Plata, Argentina; lucianoz@ciop.unlp.edu.ar; 2 Departamento de Ciencias Básicas, Facultad de Ingeniería, Universidad Nacional de La Plata (UNLP), 1900 La Plata, Argentina

**Keywords:** time series, symbolic analysis, ordinal patterns, permutation entropy, Jensen–Shannon divergence, permutation Jensen–Shannon distance, EEG records, resting brain states, multiscale analysis, linear and nonlinear temporal correlations

## Abstract

Taking into account the complexity of the human brain dynamics, the appropriate characterization of any brain state is a challenge not easily met. Actually, even the discrimination of simple behavioral tasks, such as resting with eyes closed or eyes open, represents an intricate problem and many efforts have been and are being made to overcome it. In this work, the aforementioned issue is carefully addressed by performing multiscale analyses of electroencephalogram records with the permutation Jensen–Shannon distance. The influence that linear and nonlinear temporal correlations have on the discrimination is unveiled. Results obtained lead to significant conclusions that help to achieve an improved distinction between these resting brain states.

## 1. Introduction

In the current era of big data, there is an exponential increase in observed data from complex signals, and efficient tools to manage this modern deluge of data are intensively looked for in the time series analysis community. Particularly, the brain is probably the most paradigmatic example of a complex system with an enormously large number of neurons ($∼10^{10}$) and interconnections ($∼10^{14}$) between them [1]. Emergent collective states are achieved through the synchronous neuronal activity. The popularization of different brain recording techniques, such as electroencephalography, magnetoencephalography, and functional magnetic resonance imaging, has enabled researchers to have free access to a practically unlimited amount of experimental data sets [2]. It is also true that our understanding of the functioning of the brain is quite far from being complete. Actually, despite the relevant progress achieved in recent decades, many aspects of the research in the field are still in its infancy, and it is widely recognized that to obtain a better handle on the brain dynamics is one of the most pressing, urgent and challenging issues of our time [3].

Taking into account that a more thorough comprehension of simple brain conditions is vital to better understand more complex ones, any step forward on this matter is highly appreciated. Indeed, it has previously been suggested that the brain at rest can be interpreted as a fundamental default mode and that the brain activity during a specific task may be described in terms of generic properties of the background brain activity [4]. Moreover, it has been demonstrated that the activity of the brain at rest is affected by neurological and psychiatric disorders like autism, epilepsy, Parkinson’s disease, schizophrenia, and obsessive-compulsive disorder [5,6,7,8]. In the same line, resting-state EEG measures have also been shown to be useful for the detection of cognitive decline in mild cognitive impairment and Alzheimer’s disease [9] and for decoding and predicting cognitive performance [10]. The influence of the eye’s resting condition (closed/open) on the early detection of Parkinson’s disease has been carefully tested in order to determine the most suitable biomarkers for real-time applications [11]. Likewise, better classification rates are reached in the eyes open condition in a resting state EEG for the diagnosis of Alzheimer’s disease when using permutation entropy as a neuromarker [12]. From a more practical perspective, a better knowledge of the differences between simple resting conditions, such as keeping the eyes closed (EC) and the eyes open (EO), could also be potentially useful for brain–computer interfaces. In particular, this may be helpful in enhancing switching mechanisms for assistive technologies (environmental control systems) designed to assist people with severe disabilities in daily living tasks [13].

Even when the attenuation of the alpha waves over the human posterior cortex in the EO condition is a well-known recognized fact for almost a century [14], different approaches have more recently been proposed to obtain an improved discrimination and classification of these baseline brain states [2,7,15,16,17,18,19]. It has been found, for example, that different entropy measures (permutation entropy, approximate entropy, multiscale entropy, spatial permutation entropy) achieve higher values in the EO condition compared with the EC one [2,16,18,20,21]. This can be explained by the increasing disorder and desynchronization of brain activity due to visual information receiving and processing [16]. For a similar reason, there is a differential modulation of the time-reversal symmetry by these two experimental conditions, and the time irreversibility also increases in the EO resting state with respect to the EC resting one [7].

It is also worth highlighting here the well-recognized potentiality of the ordinal pattern-based methodologies for characterizing EEG data. Without being exhaustive, the first application for detecting the onset of epileptic seizures in intracranial EEG data [22] and the foundational contributions by Li et al. [23,24] and Keller et al. [25,26] in this regard deserve to be especially mentioned. The reason for its outstanding performance likely lies in the robustness of ordinal patterns to the data artefacts and low-frequency perturbations that usually contaminate EEG records [27].

In this work, a recently introduced ordinal metric tool, the permutation Jensen–Shannon distance [28], is used to try to unravel the intricacies and interplay of linear and nonlinear temporal correlations that are inextricably present in any kind of brain activity. The results obtained prove that the implementation of the aforementioned ordinal measure on the original resting EEG records and different surrogate realizations generated from them provide relevant information about the temporal scales and spatial localizations where these linear and nonlinear components play a significant and non-negligible role. Consequently, and contrasting with previous studies on the same EEG data set, an enhanced characterization and discrimination of these simple baseline brain states is achieved.

The rest of the work is structured as follows. In Section 2, the main tool implemented along this work, namely the permutation Jensen–Shannon distance, is briefly introduced. After that, a numerical analysis is included in Section 3 to illustrate how linear and nonlinear components are unveiled through the proposed analysis. Then, in Section 4, the distinction between EC and EO resting brain states is carefully analyzed. Finally, the main findings obtained from this study and some open problems are detailed in the last Section 5.

## 2. Permutation Jensen–Shannon Distance

The permutation Jensen–Shannon distance (PJSD) is an ordinal metric introduced to quantify the degree of discernability between two arbitrary time series [28]. It has been shown that, by appropriately choosing the time series taken as a reference, a wide range of complex phenomena can be handled in a robust way. Thanks to this versatility, the PJSD represents a useful addition to the complex signals analysis methods. Moreover, this concept has very recently been generalized to evaluate the heterogeneity of multiple time series from an ordinal approach [29].

The PJSD is basically based on two constitutive ingredients: the Jensen–Shannon divergence [30] and the ordinal symbolization proposed by Bandt and Pompe (BP) [31]. The Jensen–Shannon divergence is a dissimilarity measure between two arbitrary probability distributions $P=\{p_{1},\ldots ,p_{n}\}$ and $Q=\{q_{1},\ldots ,q_{n}\}$ given by

(1) $$D_{JS}\left(P,Q\right)=S\left(\frac{P+Q}{2}\right)-\frac{1}{2}S\left(P\right)-\frac{1}{2}S\left(Q\right)\;,$$

where *S* is the Shannon entropy function, i.e., $S\left(P\right)=-\sum_{i=1}^{n}p_{i}\ln p_{i}$, and as usual, the convention 0ln0=0 is assumed in accordance with its mathematical limit. It is a quantity bounded between 0 and ln2. The minimum value is reached when the two distributions under comparison are identical while the maximum value is obtained whenever their supports are disjoints (that is, $p_{i}q_{i}=0$ for i=1,…,n). The Jensen–Shannon divergence stands out for satisfying several desirable mathematical properties: non-negativity ($D_{JS}\left(P,Q\right)\geq 0$), identity of indiscernibles ($D_{JS}\left(P,Q\right)=0$ if and only if P=Q), and symmetry ($D_{JS}\left(P,Q\right)=D_{JS}\left(Q,P\right)$) [30]. In addition, it is also well defined even when $p_{i}$ vanishes without vanishing $q_{i}$ or vice versa. More importantly, the square root of the Jensen–Shannon divergence, ${\left[D_{JS}\left(P,Q\right)\right]}^{1/2}$, is a metric since it also satisfies the triangle inequality:

(2) $${\left[D_{JS}\left(P,Q\right)\right]}^{1/2}\leq {\left[D_{JS}\left(P,R\right)\right]}^{1/2}+{\left[D_{JS}\left(R,Q\right)\right]}^{1/2},$$

with *R* a third arbitrary probability distribution [32]. Actually, more recently, it has been shown that ${\left[D_{JS}\left(P,Q\right)\right]}^{\alpha }$ for α∈(0,1/2] is also a metric [33]. Extensions of this divergence to weigh differently the compared distributions and/or to quantify the overall difference among any finite number of probability distributions are also available. Interpretations and statistical properties of the Jensen–Shannon divergence have been exhaustively analyzed by Grosse et al. [34].

The estimation of the Jensen–Shannon distance, ${\left[D_{JS}\left(P,Q\right)\right]}^{1/2}$, between two time series requires first to know the corresponding probability distributions *P* and *Q* associated with the two time series under analysis. It is, therefore, necessary to map continuous time series into discrete sequences. For such a purpose, the BP coarse-grained representation [31], introduced more than 20 years ago, is implemented. This symbolization methodology has been successfully applied in heterogeneous fields. The main reasons behind this success are its wide applicability and the fact that the BP discretization method is naturally able to capture the presence of underlying temporal correlations in the dynamics of the process that generates the time series. Interested readers are referred to the reviews [35,36,37,38,39,40] for further details. The BP recipe for symbolizing a time series can be briefly summarized as follows. Given a real-valued time series $\left\{x_{t}\in R,t=1,\ldots ,L\right\}$, vectors of equally spaced *D* values of the form $\left(x_{s},x_{s+\tau },\ldots ,x_{s+(D-1)\tau }\right)$ with $s=1,\ldots ,L^{*}=L-\left(D-1\right)\tau$ are mapped to one of the D! possible ordinal permutations of the same size that describe the order relation between these elements. For example, the vector (2.5,3.7,1.2) is mapped to the permutation pattern (2,3,1), replacing each element in the original vector by its respective ranking in the subset. Assigning a symbol $\pi_{i}$ to each ordinal pattern, the original time series is mapped to the coarse-graining sequence $\left\{y_{s}\in \Pi_{D},s=1,\ldots ,L^{*}\right\}$, with $\Pi_{D}=\left\{\pi_{1},\pi_{2},\ldots ,\pi_{D!}\right\}$ the set of permutations of length *D*. Just for illustrative purpose, $\Pi_{3}=\{\pi_{1}=\left(1,2,3\right),$ $\pi_{2}=\left(1,3,2\right),$ $\pi_{3}=\left(2,1,3\right),\pi_{4}=\left(2,3,1\right),\pi_{5}=\left(3,1,2\right),\pi_{6}=\left(3,2,1\right)\}$. There are therefore two parameters which need to be fixed before the method is implemented: the number of elements in the permutation patterns *D* (called order or embedding dimension, D≥2 with D∈N) and the time separation between the elements in the subsequence τ (called lag or embedding delay, τ∈N). Consecutive data are considered if τ=1, while τ−spaced data samples are analyzed if τ≥2. An associated ordinal probability distribution,

(3) $$P_{\pi }^{D,\tau }=\left\{p\left(\pi_{i}\right),i=1,\ldots ,D!\right\},$$

can then be straightforwardly computed with $p(\pi_{i})$ the probability of each ordinal pattern estimated by its relative frequency of occurrence in the symbolized sequence. Regarding the selection of the involved parameters, a rule of thumb requires the condition L≫D!, with *L* the number of data in the original time series, for a robust estimation of $P_{\pi }^{D,\tau }$. It is also clear that larger values of *D* offer an improved characterization of the underlying temporal structures. On the other hand, a value of the lag τ=1 is often used in discrete systems and also when the chosen sampling frequency is the optimal one to characterize the underlying dynamics of continuous systems [38]. However, this arbitrary choice can lead to erroneous conclusions especially for systems with scale-dependent dynamics [41]. A multiscale analysis, by analyzing how descriptors of the ordinal distribution change with τ, gives a more complete picture in these instances [42]. Finally, a word of caution when the number of equalities in the time series is non-negligible. Since the BP recipe is based on sorted data, it is needed to clarify how they will be handled. This is particularly relevant when dealing with low-resolution time series. Regarding this issue, it has been shown that the estimation of the ordinal probability distribution could be significantly biased in such cases, and that the addition of a small random perturbation mitigates this unwanted effect [43].

Different statistics can be computed from the resulting permutation probability distribution given in Equation (3). The permutation entropy (PE) [31], which is just defined as the Shannon entropy of this ordinal distribution,

(4) $$S\left(P_{\pi }^{D,\tau }\right)=-\sum_{i=1}^{D!}p\left(\pi_{i}\right)\log p\left(\pi_{i}\right),$$

is undoubtedly the most representative and widely used descriptor. It quantifies the variety of permutation patterns in the ordinal sequence obtained from a time series. The maximum value, $S_{\max}=\log D!$, is obtained for a totally random stochastic process (white noise), while the minimum value, $S_{\min}=0$, is reached for a completely regular (monotonically increasing or decreasing) time series. The normalized permutation entropy $H=S\left(P_{\pi }^{D,\tau }\right)/S_{\max}$, such that 0≤H≤1, is often implemented in practical analysis.

The PJSD is defined as the Jensen–Shannon distance between the ordinal pattern distributions associated with two arbitrary time series, ${\left[D_{JS}\left(P_{\pi }^{D,\tau_{1}},Q_{\pi }^{D,\tau_{2}}\right)\right]}^{1/2}$. More precisely, normalized PJSD values with respect to its upper bound, i.e., ${\left(\log2\right)}^{1/2}$, are estimated. It can be used to compare the ordinal mapping between different time series. Numerical and real data applications included in Ref. [28] confirm its utility for characterizing, classifying and discriminating different dynamics from the analysis of time series generated by them. Moreover, its robustness to noise and invariance under scaling of the data make this ordinal distance especially appropriate for the analysis of experimental signals. When calculating the PJSD, the order *D* chosen to implement the BP symbolization recipe should be the same for both time series in order to have the same number of possible permutation patterns in the ordinal probability distributions to be compared. However, different lags ($\tau_{1}$ and $\tau_{2}$) can be potentially selected. This opens up the possibility to contrast the ordinal mappings of only one or two time series at two different temporal scales.

## 3. Numerical Analysis

Trying to illustrate how the PJSD can be implemented to unveil linear and nonlinear underlying dynamics from a time series, a numerically controlled analysis on a toy model is developed below. More precisely, realizations of the logistic map in the fully chaotic regime, a paradigmatic example of a nonlinear complex system, are mixed with simulations from a linear first-order autoregressive AR(1) process according to the following model:

(5) $$z_{n}=mx_{n}+\left(1-m\right)y_{n}$$

with $x_{n}=4x_{n-1}\left(1-x_{n-1}\right)$, $y_{n}=\alpha y_{n-1}+\epsilon_{n}$ ($\epsilon_{t}$ are pseudorandom values drawn from the standard normal distribution), and *m* is the fraction of nonlinear dynamics included in the mixed time series (0≤m≤1). When m=0 and m=1, the pure AR(1) and logistic models are recovered, respectively, while as *m* goes from 0 to 1, the mixed time series include less linear stochastic and more nonlinear deterministic structures. The parameter α of the AR(1) model should satisfy the condition |α|<1 to have a stationary process, and larger correlations and anti-correlations are realized as this parameter moves away from zero in the positive and negative direction, respectively. The parameter α is fixed to be equal to 0.9 in the present analysis. A similar, but not exactly the same, mixed model has previously been proposed to evaluate the performance of network tools in statistical tests to detect weak nonlinearities [44].

Next, surrogate data sets are generated to extract information about the linear and/or nonlinear nature of the original time series. Particularly, shuffled and Fourier transform based surrogates are considered in the performed analysis. On the one hand, in the first generating algorithm, also known as scrambled or random permutation, the surrogate time series is conducted by shuffling the time order of the original time series and, consequently, any underlying temporal structure (linear and/or nonlinear) is destroyed. These randomized resampled sequences are fully random data that have exactly the same amplitude distribution as the original time series, and the associated null hypothesis is that the data under analysis are just uncorrelated noise with an arbitrary amplitude distribution. On the other hand, with the Fourier transform (FT)-based algorithm, a data sequence with the same autocorrelation function as the original time series is created by randomizing the phases of the discrete Fourier transform of the original time series and then computing the inverse transform [45]. In this way, the linear behavior contained in the original data are preserved, while the nonlinear temporal structure is fully removed. The null hypothesis is hence that the data that come from a linear Gaussian process. Some generalizations of the FT surrogate generation method, such as the amplitude adjusted Fourier transform (AAFT) and iterative amplitude adjusted Fourier transform (IAAFT), seek to reproduce both the autocorrelation function and the amplitude distribution of the original data [46]. Interested readers are referred to Ref. [47] for a thorough review on surrogate data testing.

By estimating the PJSD between the primary time series and its shuffled surrogate counterpart, the influence that any kind of temporal correlation (linear and nonlinear) has on the system that generates the original data can be directly measured. Similarly, the PJSD between the original time series and its FT surrogate quantifies the relevance of the role played by nonlinearity on the underlying system dynamics. Finally, doing the same, i.e., estimating the ordinal distance, but between the two surrogate realizations (shuffled and FT), allows us to quantify the impact that linearity, solely, has on the dynamics. Thus, these three surrogate-based ordinal distances offer a practical and easy way to identify and quantify the linear and/or nonlinear nature of a system from the analysis of a time series generated by it.

**Figure 1:**
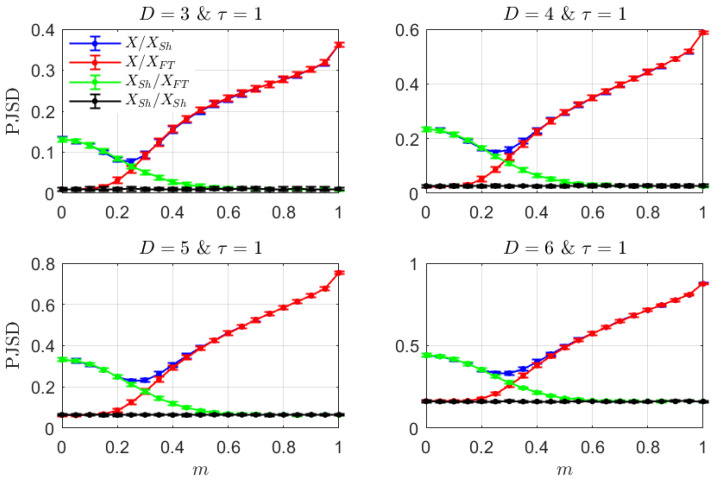
Mean and standard deviation (as error bar) of the PJSD estimations with D∈{3,4,5,6} and τ=1 from an ensemble of 100 independent realizations with length $L=10^{4}$ data for the mixed model with parameter α=0.9. $\mathrm{PJSD}(P_{X},P_{X_{Sh}})$ (blue curve), $\mathrm{PJSD}(P_{X},P_{X_{FT}})$ (red curve), $\mathrm{PJSD}(P_{X_{Sh}},P_{X_{FT}})$ (green curve), and $\mathrm{PJSD}(P_{X_{Sh}},P_{X_{Sh}})$ (black curve) are plotted as a function of the fraction *m* (m∈{0,0.05,0.1,…,0.95,1}). The first ordinal distance characterizes linear and nonlinear behaviors globally, the second one quantifies just the nonlinear component, the third one measures linear temporal correlations solely, and, finally, the last ordinal distance is estimated to set a baseline reference. Results obtained are qualitatively analogous for the different orders *D*.

Figure 1 shows the results obtained when applying the previously described analysis to the simple mixed model defined by Equation (5). Mean and standard deviation (as an error bar) of the PJSD estimations with D∈{3,4,5,6} and $\tau_{1}=\tau_{2}=\tau =1$ from an ensemble of 100 independent realizations with length $L=10^{4}$ data for the mixed model with α=0.9 are depicted as a function of the fraction *m*. The choice of the lag parameter is plenty justified by the discrete nature of the toy numerical model. It is important to note that in the simulation process, both involved signals in the mixed model, i.e., $x_{n}$ and $y_{n}$, have been standardized (mean equal to 0 and standard deviation equal to 1) previously to be added, in order to make the comparison between both dynamics more equitable. The estimated ordinal distances behave consistently when they are plotted as a function of the fraction of nonlinearity *m* (*m* goes from 0 to 1 with step 0.05). That is, the PJSD between the original time series and its shuffled surrogate, $\mathrm{PJSD}(P_{X},P_{X_{Sh}})$ (blue curve), that quantifies both, linear and nonlinear, structures, decreases with *m* up to reach a minimum value around *m*∼0.25–0.3. At this particular fraction value, it is possible to conjecture that there is a maximum interplay between the linear and nonlinear natures occurring in the mixed model. After it, the PJSD increases monotonically due to the growing impact of the deterministic nonlinear dynamics, while the other two proposed ordinal distances that characterize nonlinear and linear components, i.e., $\mathrm{PJSD}(P_{X},P_{X_{FT}})$ (red curve) and $\mathrm{PJSD}(P_{X_{Sh}},P_{X_{FT}})$ (green curve), increases and decreases monotonically with *m*, respectively, as was expected. Moreover, these two curves overlap consistently with the more general one that contemplates the effect of both components: $\mathrm{PJSD}\left(P_{X},P_{X_{FT}}\right)\approx \mathrm{PJSD}\left(P_{X},P_{X_{Sh}}\right)$ for *m* larger than 0.35, whereas $\mathrm{PJSD}\left(P_{X_{Sh}},P_{X_{FT}}\right)\approx \mathrm{PJSD}\left(P_{X},P_{X_{Sh}}\right)$ when *m* is smaller or equal than 0.2. According to these findings, the linear stochastic and nonlinear deterministic components govern the dynamics of the mixed model in the ranges [0,0.2] and [0.4,1] of *m*, respectively. A transition region, where the effect of both components seems to be weakened, happens in the intermediate range. The PJSD baseline reference resulting from the analysis of a pair of shuffled surrogate realizations from each model simulation, $\mathrm{PJSD}(P_{X_{Sh}},P_{X_{Sh}})$ (black curve), has also been included to take finite-size effects into consideration. It is clearly evidenced that there exists a systematic shift or bias that depends on the order *D*.

**Figure 2:**
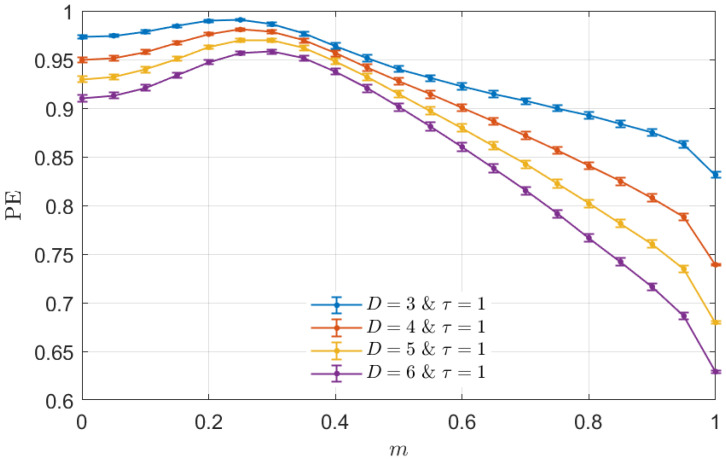
Mean and standard deviation (as error bar) of the PE estimations with D∈{3,4,5,6} and τ=1 from the same ensemble of numerical realizations of the mixed model detailed in Figure 1. The behavior followed by the PE as a function of the fraction *m* is opposed to that observed for $\mathrm{PJSD}(P_{X},P_{X_{Sh}})$. Thus, both measures provide the same qualitative information about the mixed model.

Just for the sake of completeness, and taking into account that the PE is the most representative and established descriptor within the ordinal encoding recipe, Figure 2 shows the behavior of this entropic measure for the mixed model as a function of the parameter *m*. Once again, mean and standard deviation (as error bars) for PE estimations with D∈{3,4,5,6} and τ=1 from the same ensemble of 100 independent realizations for each value of *m* are depicted. By comparing Figure 1 and Figure 2, it can be concluded that the behavior observed for PE is opposed to the one obtained for $\mathrm{PJSD}(P_{X},P_{X_{Sh}})$, achieving a maximum where the latter reaches a minimum, and increasing and decreasing in the same range of *m* in which the ordinal distance between the original time series and its shuffled surrogate decreases and increases, respectively. Essentially, PE decreases when linear and/or nonlinear temporal correlations are included in the dynamics. Consequently, PE and $\mathrm{PJSD}(P_{X},P_{X_{Sh}})$ offer the same qualitative information when they are used to examine the proposed mixed model. Therefore, PE quantifies the presence of any kind of temporal correlation in a global sense, and when implementing it as a statistic, it is not directly feasible to discriminate between the linear and nonlinear components occurring in the system dynamics.

Previous analysis has been repeated for other values of the correlation parameter α and, also, for the two aforementioned improved FT surrogate methods (AAFT and IAAFT). Results obtained are qualitatively and quantitatively similar in the former and latter cases, respectively. It is worth noting that, when considering other α values, the ordinal distances that take linear correlations into account ($\mathrm{PJSD}(P_{X},P_{X_{Sh}})$ and $\mathrm{PJSD}(P_{X_{Sh}},P_{X_{FT}})$) change accordingly. That is, they increase for low values of *m* as α moves from 0 to 1.

## 4. Resting Brain States Characterization

EEG data from 109 healthy subjects in two different states: resting with their eyes closed and resting with their eyes open have been analyzed. These data are freely available from PhysioNet [48], and they include a set of 64-channel EEGs sampled at 160 samples per second from subjects who performed a series of different motor/imagery tasks [49], including the two baseline runs of resting state conditions, both recorded during a period of one minute. The EEGs were recorded from 64 electrodes according to the international 10-10 system. Please see https://physionet.org/content/eegmmidb/1.0.0/ (accessed on 31 March 2024) for further details. It is worth remembering here that EEG is a noninvasive and portable technique able to capture brain electric activity with high temporal resolution.

It is well known that the brain is a multiscale system in which typical rhythmic patterns are located at specific regions. Actually, a detailed atlas of the natural frequencies of the human brain at rest have recently been developed [50]. Bearing in mind this inherent spatio-temporal architecture of the resting human brain, a PJSD analysis per channel varying the lag τ, i.e., at different time scales, has been performed. The main goal is to simultaneously look for brain regions and temporal scales that maximize the discrimination between the two resting states (EC and EO). Moreover, trying to disentangle the influence that linear and nonlinear temporal correlations have on the discriminative power, an analysis analogous to the one detailed in the previous section has been carried out.

**Figure 3:**
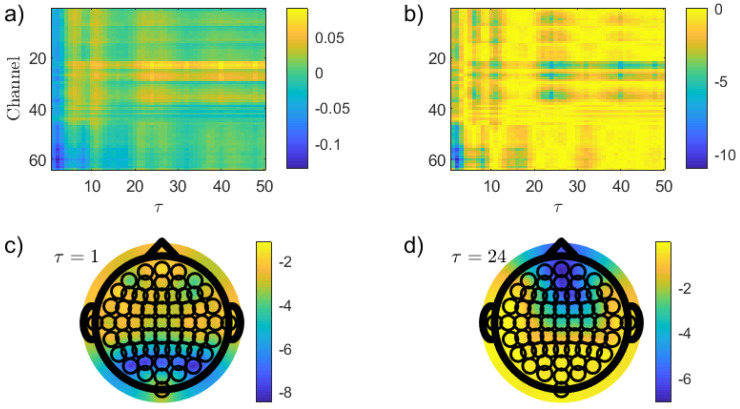
Discrimination of the two resting states, EO and EC, by implementing $\mathrm{PJSD}(P_{X},P_{X_{Sh}})$ as discriminative statistic. Results obtained for the raw EEG records when an order D=6 is chosen for estimating the ordinal quantifier are depicted. Differences in average between EO and EC resting conditions and *p*-values (in logarithmic base 10 scale), calculated by using the Wilcoxon rank sum test, as a function of the channel and the lag τ (τ∈{1,2,…,50}) are displayed in (**a**,**b**), respectively. Topographic maps of the *p*-values (in logarithmic base 10 scale) for two specific lags, τ=1 and τ=24, are illustrated in (**c**,**d**), respectively.

Figure 3 shows the results obtained when the $\mathrm{PJSD}(P_{X},P_{X_{Sh}})$ is implemented to discriminate between the EC and EO resting states. Differences in average between the EO and EC conditions, $\langle \mathrm{PJSD}\left(P_{X},P_{X_{Sh}}\right){|}_{EO}\rangle$−$\langle \mathrm{PJSD}\left(P_{X},P_{X_{Sh}}\right){|}_{EC}\rangle$, as a function of the channel and lag τ ($\tau_{1}=\tau_{2}=\tau \in \left\{1,2,\ldots ,50\right\}$) are depicted in Figure 3a while *p*-values (in logarithmic base 10 scale), calculated by using the Wilcoxon rank sum test, are shown in Figure 3b. This non-parametric approach can be used to identify significant differences between two independent samples. The Matlab code *ranksum* was implemented for such a purpose (Matlab Version 9.5, R2018b). For further details, interested readers are referred to https://www.mathworks.com/help/stats/ranksum.html (accessed on 31 March 2024). Topographic visualizations of these *p*-values in logarithmic base 10 scale for two specific lags, τ=1 and τ=24, are displayed in Figure 3c and Figure 3d, respectively. Several interesting conclusions can be drawn from these plots. First, the optimal discrimination between the EO and EC states are obtained for the occipital electrodes when the EEG records are mapped with a lag τ=2, i.e., at a sampling time very close to the original one (τ=1). This finding is clearly not surprising in view of the fact that the primary visual cortex is located in the occipital lobe [2,16]. Additionally, $\mathrm{PJSD}(P_{X},P_{X_{Sh}})$ is lower in the EO than in the EC condition for low values of the lag τ. Putting this in another way, temporal correlations (linear and/or nonlinear) are stronger in the latter resting condition for these temporal scales. However, the situation changes drastically when larger values of lag (smaller sampling times) are considered since frontal electrodes achieve the lowest *p*-values for these temporal scales with larger values of the quantifier in the EO resting state. This is a much more novel and appealing result, which could be potentially correlated with the critical role played by the frontal lobe in visual perception [16,51]. Further tests are obviously required to validate this hypothesis. On the other hand, it is also important to highlight that the optimal discrimination powers in the frontal channels are not as high as the one obtained for the occipital ones.

In order to check if the well-known stronger alpha activity in the eye-closed condition plays a main role in the previously obtained result, a simple numerical analysis has been developed. A sine wave with a frequency of 10 Hz was sampled at a sampling rate of 160 Hz and an appropriate fraction of random numbers from a standard normal distribution was then added to simulate the noise stochastic component. It is worth remembering here that alpha waves have frequencies from 8 to 12 Hz, approximately. After performing the same analysis by using $\mathrm{PJSD}(P_{X},P_{X_{Sh}})$ as a discriminative statistic between the simulated noisy oscillations and fully random dynamics, it has been found that the optimal lag scales that minimize the *p*-value are τ=8, τ=24 and τ=40. They coincide with the odd multiples of the half-period of the sinusoidal waveform. Actually, the improved recognition of periodicities from periodic dynamics at these particular values of the lag τ has been carefully confirmed by Bandt by using ordinal autocorrelation functions [52]. Thus, those are the lag scales in which the alpha rhythm is better resolved, and they consequently optimize the discrimination between the resting states (please see Figure 3b). There are, however, other lags for which the distinction is enhanced, and the explanation behind this better discrimination is not as direct and immediate. Further research that is beyond the scope of this study is needed to fully understand them.

**Figure 4:**
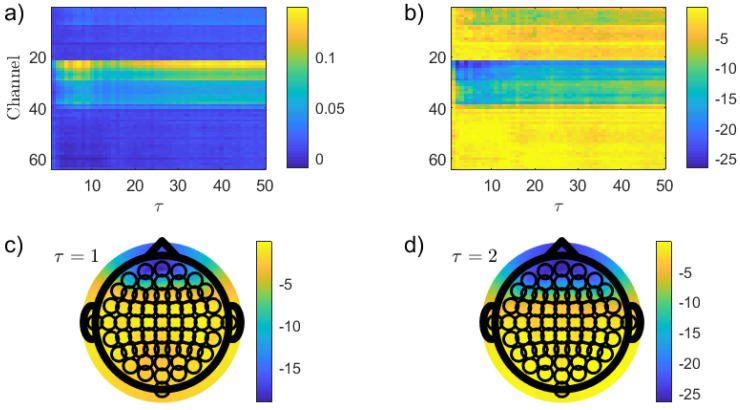
The same as in Figure 3 but using $\mathrm{PJSD}(P_{X},P_{X_{FT}})$ as the discriminative statistic. In this case, the topographic map of the *p*-values (in logarithmic base 10 scale) for τ=2 is illustrated in (**d**).

With the intention to explore the impact that solely nonlinear temporal correlations have on the distinction between the two resting states, an analogous analysis using the $\mathrm{PJSD}(P_{X},P_{X_{FT}})$ has been developed. Figure 4 shows the results obtained in this case. Frontal channels have more intense nonlinear correlations in the EO than in the EC resting condition (Figure 4a), and an enhanced discrimination is found in this brain region practically for all τ values under consideration (Figure 4b). The lowest *p*-values, reached for for τ=2 (Figure 4d), are several orders of magnitude lower than those estimated in the previous analysis for $\mathrm{PJSD}(P_{X},P_{X_{Sh}})$. This result is of high relevance just because a much more robust, spatially located discrimination of the resting states is observed. Even when the reasons behind the presence of stronger nonlinear signatures in the frontal channels for the EO condition are not clear at all and that an interpretation from a neurophysiological perspective is highly necessary, leveraging on this heuristic finding, further insights can be achieved.

**Figure 5:**
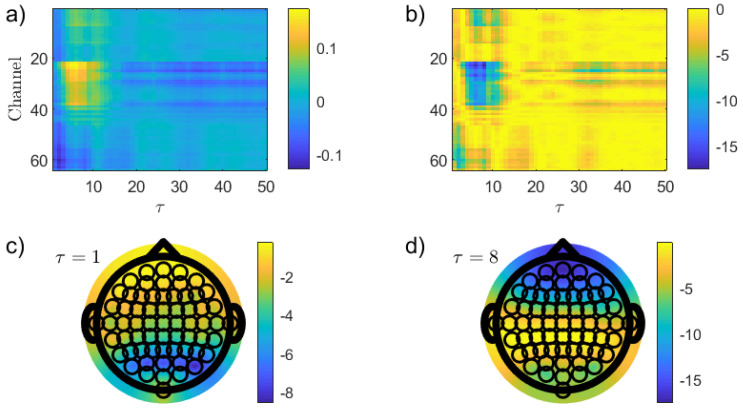
The same as in Figure 3 but using $\mathrm{PJSD}(P_{X_{Sh}},P_{X_{FT}})$ as a discriminative statistic. In this case, the topographic map of the *p*-values (in logarithmic base 10 scale) for τ=8 is illustrated in (**d**).

Analogously, $\mathrm{PJSD}(P_{X_{Sh}},P_{X_{FT}})$ has been implemented as a discriminative statistic to unveil the role played by linear temporal correlations (solely) in the EO vs. EC conditions. The main findings are displayed in Figure 5. It is found that channels in the frontal region of the brain with lag τ∈[5,10] have the highest discrimination powers. Under these circumstances, linear correlations in the EO resting state are stronger than those in the EC one. It is also worth noting that this behavior changes in the opposite way, i.e., the EO condition has weaker linear correlations than in the EC condition, for occipital and some particular frontal channels at small and large τ values, respectively. Indeed, significant differences between both resting conditions are also achieved in such cases, but the associated *p*-values are not the smallest ones.

**Figure 6:**
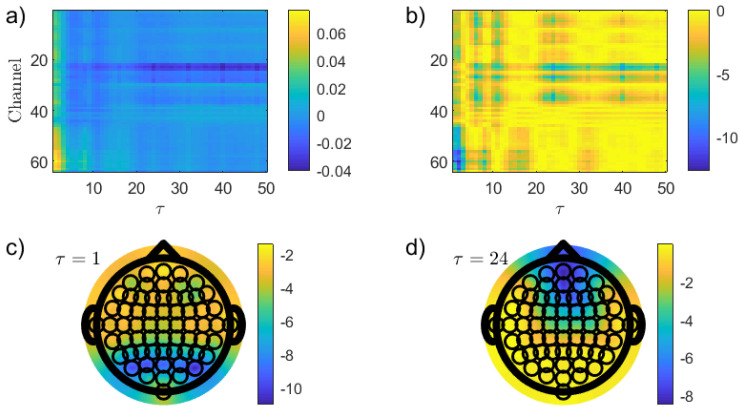
The same as in Figure 3 but using PE as discriminative statistic.

Finally, and for comparison purposes, Figure 6 illustrates the results obtained when PE is estimated from the resting EEG raw data. On the one hand, it is found that the behavior observed when using the PE as discriminative statistic is strongly correlated with that found for the $\mathrm{PJSD}(P_{X},P_{X_{Sh}})$ (Figure 3), attaining very similar levels of discrimination in both cases. Once again, as it happens in the numerical analysis developed in Section 3, both measures, PE and $\mathrm{PJSD}(P_{X},P_{X_{Sh}})$, give essentially the same qualitative information. On the other hand, by comparing with Figure 4 and Figure 5, it is clear that $\mathrm{PJSD}(P_{X},P_{X_{FT}})$ and $\mathrm{PJSD}(P_{X_{Sh}},P_{X_{FT}})$ are able to provide useful additional information about the linear and/or nonlinear temporal structures occurring in the brain resting dynamics. Moreover, temporal scales and regions of the brain where these components undertake a relevant role are more robustly identified.

Throughout all of this section, results obtained for the original EEG records with order D=6 are illustrated. However, it is worth remarking here that very similar findings are obtained for other orders (D=3, D=4 and D=5), and also when a tiny random perturbation is added to the raw EEGs for breaking equalities. Moreover, it has also been checked that obtained *p*-values are qualitatively analogous when other statistical tests, such as the two-sample *t*-test or the Welch’s *t*-test, are applied.

It is clear that the comparison with other nonlinear techniques would be very useful to evaluate the advantages of the proposed methodology in a more thorough way. Furthermore, in recent years, more powerful ordinal tools have been introduced, such as the generalized statistical complexity measure based on ordinal transition frequencies instead of ordinal pattern frequencies [53] and some time irreversibility metrics based on permutation patterns [54], that can help to reach an enhanced performance in the discrimination task. However, the results included in this section confirm that the $\mathrm{PJSD}(P_{X},P_{X_{FT}})$ and $\mathrm{PJSD}(P_{X_{Sh}},P_{X_{FT}})$ offer improved discrimination powers than the PE, which is the most widely used ordinal measure, by several orders of magnitude. It is then quite reasonable to conjecture that the multiscale surrogate approach by implementing the PJSD could overcome some very recently observed limitations of the PE for distinguishing eyes-open and eyes-closed brain states when the same dataset is analyzed [55].

## 5. Conclusions

It is unquestionable that spontaneous electroencephalographic brain activity carries relevant information that can help us to decode complex brain functioning. Hence, a robust characterization of resting states is a challenge that deserves special consideration and efforts. In the present work, the focus has been put on discriminating between the EC and EO states from EEG data of healthy subjects. An improved characterization of these two apparently trivial brain conditions is a fundamental step towards a better understanding of more demanding cognitive tasks. By implementing an approach based on the PJSD, temporal scales and brain channels that optimize the distinction between these two baseline resting states have been revealed.

Main conclusions obtained from the analyses developed in this work are listed below:

- It is shown that $\mathrm{PJSD}(P_{X},P_{X_{Sh}})$ and PE offer equivalent information. Both measures quantify linear and nonlinear correlations in a global way. When they are used as a discriminative tool, occipital and frontal electrodes at low and intermediate/large τ values, respectively, reach the highest discrimination powers;
- $\mathrm{PJSD}(P_{X},P_{X_{FT}})$ unveils the role played by nonlinear correlations solely. When applied to the EO vs. EC problem, it is found that frontal channels at practically all τ values under consideration (1≤τ≤50) achieve the most significant differences;
- $\mathrm{PJSD}(P_{X_{Sh}},P_{X_{FT}})$ characterizes the impact that the presence of linear correlations have on the dynamics. Frontal channels with lag τ∈[5,10] have the highest discrimination powers when it is implemented as a quantifier for discriminating the resting EEG data;
- The lowest *p*-values are obtained for $\mathrm{PJSD}(P_{X},P_{X_{FT}})$ in the frontal channels. The presence of more intense nonlinear correlations in the EO resting condition than in the EC one in the frontal lobe is the justification for them;
- The differences observed between the baseline resting conditions are much more significant, i.e., the associated *p*-values are several orders of magnitude lower, when linear and nonlinear correlations are independently considered by estimating $\mathrm{PJSD}(P_{X_{Sh}},P_{X_{FT}})$ and $\mathrm{PJSD}(P_{X},P_{X_{FT}})$, respectively;
- When contrasted with the results obtained by using the PE, which is the most widely accepted ordinal benchmark, it is found that the proposed multiscale surrogate methodology by estimating the PJSD offers enhanced discrimination powers specially for channels located in the frontal brain area. It is important to stress that this novel heuristic finding necessarily deserves further neurophysiological interpretations. This issue should be addressed in future research;
- Finally, it is important to warn that the success of the proposed methodology strongly depends on the appropriate generation of surrogate realizations. In particular, generating reliable surrogates which only mimic the linear properties of the original data represents a subtle issue, not easily tackled. The presence of spurious static and/or dynamic nonlinearities in the supposedly pure linear surrogates could definitely lead to wrong conclusions [56]. Hence, it would be important to try to confirm the findings achieved in the resting EEG analysis by using a different approach, not based on surrogate data sets. This clearly constitutes another very interesting issue to try to deal with.

Reaching an improved understanding of the underlying mechanisms that govern the brain dynamics at rest is a fundamental step prior to building more suitable theoretical brain models. Results obtained evidence that the analysis proposed in this work is a powerful tool in such a context. It could also be particularly useful for identifying and detecting potential biomarkers of neurological disorders, and, consequently, it may also provide important insights about the success of different kinds of treatment strategies. Furthermore, it is reasonable to hypothesize that other scientific fields could also be significantly benefited by its implementation. Hence, interested researchers are strongly encouraged to use this procedure in their field of interest in order to obtain a more comprehensive understanding of its scope and limitations. 

## Data Availability

The EEG data analyzed in this study are publicly available from PhysioNet at https://physionet.org/content/eegmmidb/1.0.0/ (accessed on 31 March 2024).

## Abbreviations

The following abbreviations are used in this manuscript:

EEG	Electroencephalogram
EC	Eyes Closed
EO	Eyes Open
PJSD	Permutation Jensen–Shannon Distance
BP	Bandt and Pompe
PE	Permutation Entropy
FT	Fourier Transform
AR(1)	First-order Autoregressive Process
AAFT	Amplitude Adjusted Fourier Transform
IAAFT	Iterative Amplitude Adjusted Fourier Transform

## References

[1] Kwapień J., Drożdż S. (2012). Physical approach to complex systems. Phys. Rep..

[2] Quintero-Quiroz C., Montesano L., Pons A.J., Torrent M.C., García-Ojalvo J., Masoller C. (2018). Differentiating resting brain states using ordinal symbolic analysis. Chaos Interdiscip. J. Nonlinear Sci..

[3] Bianconi G., Arenas A., Biamonte J., Carr L.D., Kahng B., Kertesz J., Kurths J., Lü L., Masoller C., Motter A.E. (2023). Complex systems in the spotlight: Next steps after the 2021 Nobel Prize in Physics. J. Phys. Complex..

[4] Papo D. (2013). Why should cognitive neuroscientists study the brain’s resting state?. Front. Hum. Neurosci..

[5] Pérez Velázquez J.L., Galán R. (2013). Information gain in the brain’s resting state: A new perspective on autism. Front. Neuroinform..

[6] Yi G.S., Wang J., Deng B., Wei X.L. (2017). Complexity of resting-state EEG activity in the patients with early-stage Parkinson’s disease. Cogn. Neurodyn..

[7] Zanin M., Güntekin B., Aktürk T., Hanoğlu L., Papo D. (2020). Time irreversibility of resting-state activity in the healthy brain and pathology. Front. Physiol..

[8] Bernardi D., Shannahoff-Khalsa D., Sale J., Wright J.A., Fadiga L., Papo D. (2023). The time scales of irreversibility in spontaneous brain activity are altered in obsessive compulsive disorder. Front. Psychiatry.

[9] Meghdadi A.H., Karić M.S., McConnell M., Rupp G., Richard C., Hamilton J., Salat D., Berka C. (2021). Resting state EEG biomarkers of cognitive decline associated with Alzheimer’s disease and mild cognitive impairment. PLoS ONE.

[10] Wan W., Gao Z., Zhang Q., Gu Z., Chang C., Peng C.K., Cui X. (2023). Resting state EEG complexity as a predictor of cognitive performance. Phys. A Stat. Mech. Its Appl..

[11] Gimenez-Aparisi G., Guijarro-Estelles E., Chornet-Lurbe A., Ballesta-Martinez S., Pardo-Hernandez M., Lin Y.Y. (2023). Early detection of Parkinson’s disease: Systematic analysis of the influence of the eyes on quantitative biomarkers in resting state electroencephalography. Heliyon.

[12] Şeker M., Özbek Y., Yener G., Özerdem M.S. (2021). Complexity of EEG dynamics for early diagnosis of Alzheimer’s disease using permutation entropy neuromarker. Comput. Methods Programs Biomed..

[13] Thuraisingham R.A., Tran Y., Boord P., Craig A. (2017). Analysis of eyes open, eye closed EEG signals using second-order difference plots. Med. Bio. Eng. Comput..

[14] Barry R.J., Clarke A.R., Johnstone S.J., Magee C.A., Rushby J.A. (2007). EEG differences between eyes-closed and eyes-open resting conditions. Clin. Neurophysiol..

[15] Olejarczyk E., Marzetti L., Pizzella V., Zappasodi F. (2017). Comparison of connectivity analyses for resting state EEG data. J. Neural Eng..

[16] Vecchio F., Miraglia F., Pappalettera C., Orticoni A., Alù F., Judica E., Cotelli M., Rossini P.M. (2021). Entropy as measure of brain networks’ complexity in eyes open and closed conditions. Symmetry.

[17] Khosla A., Khandnor P., Chand T. (2022). A novel method for EEG based automated eyes state classification using recurrence plots and machine learning approach. Concurr. Comput. Pract. Exp..

[18] Boaretto B.R., Budzinski R.C., Rossi K.L., Masoller C., Macau E.E. (2023). Spatial permutation entropy distinguishes resting brain states. Chaos Solitons Fractals.

[19] Restrepo J.F., Mateos D.M., López J.M.D. (2023). A Transfer entropy-based methodology to analyze information flow under eyes-open and eyes-closed conditions with a clinical perspective. Biomed. Signal Process. Control.

[20] Ricci L., Perinelli A. (2022). Estimating permutation entropy variability via surrogate time series. Entropy.

[21] Walter N., Hinterberger T. (2022). Determining states of consciousness in the electroencephalogram based on spectral, complexity, and criticality features. Neurosci. Conscious..

[22] Cao Y., Tung W.W., Gao J.B., Protopopescu V.A., Hively L.M. (2004). Detecting dynamical changes in time series using the permutation entropy. Phys. Rev. E.

[23] Li X., Ouyang G., Richards D.A. (2007). Predictability analysis of absence seizures with permutation entropy. Epilepsy Res..

[24] Ouyang G., Li X., Dang C., Richards D.A. (2009). Deterministic dynamics of neural activity during absence seizures in rats. Phys. Rev. E.

[25] Keller K., Unakafov A.M., Unakafova V.A. (2014). Ordinal patterns, entropy, and EEG. Entropy.

[26] Keller K., Mangold T., Stolz I., Werner J. (2017). Permutation entropy: New ideas and challenges. Entropy.

[27] Bandt C. (2017). A new kind of permutation entropy used to classify sleep stages from invisible EEG microstructure. Entropy.

[28] Zunino L., Olivares F., Ribeiro H.V., Rosso O.A. (2022). Permutation Jensen-Shannon distance: A versatile and fast symbolic tool for complex time-series analysis. Phys. Rev. E.

[29] Zunino L., Soriano M.C. (2023). Quantifying the diversity of multiple time series with an ordinal symbolic approach. Phys. Rev. E.

[30] Lin J. (1991). Divergence measures based on the Shannon entropy. IEEE Trans. Inf. Theory.

[31] Bandt C., Pompe B. (2002). Permutation entropy: A natural complexity measure for time series. Phys. Rev. Lett..

[32] Endres D., Schindelin J. (2003). A new metric for probability distributions. IEEE Trans. Inf. Theory.

[33] Osán T.M., Bussandri D.G., Lamberti P.W. (2018). Monoparametric family of metrics derived from classical Jensen–Shannon divergence. Phys. A Stat. Mech. Its Appl..

[34] Grosse I., Bernaola-Galván P., Carpena P., Román-Roldán R., Oliver J., Stanley H.E. (2002). Analysis of symbolic sequences using the Jensen-Shannon divergence. Phys. Rev. E.

[35] Zanin M., Zunino L., Rosso O.A., Papo D. (2012). Permutation entropy and its main biomedical and econophysics applications: A review. Entropy.

[36] Amigó J.M., Keller K., Kurths J. (2013). Recent progress in symbolic dynamics and permutation complexity—Ten years of permutation entropy. Eur. Phys. J. Spec. Top..

[37] Amigó J.M., Keller K., Unakafova V.A. (2015). Ordinal symbolic analysis and its application to biomedical recordings. Phil. Trans. R. Soc. A.

[38] Zanin M., Olivares F. (2021). Ordinal patterns-based methodologies for distinguishing chaos from noise in discrete time series. Commun. Phys..

[39] Leyva I., Martínez J.H., Masoller C., Rosso O.A., Zanin M. (2022). 20 years of ordinal patterns: Perspectives and challenges. Europhys. Lett..

[40] Amigó J.M., Rosso O.A. (2023). Ordinal methods: Concepts, applications, new developments, and challenges—In memory of Karsten Keller (1961–2022). Chaos.

[41] Olivares F., Zunino L. (2020). Multiscale dynamics under the lens of permutation entropy. Phys. A Stat. Mech. Its Appl..

[42] Zunino L., Soriano M.C., Rosso O.A. (2012). Distinguishing chaotic and stochastic dynamics from time series by using a multiscale symbolic approach. Phys. Rev. E.

[43] Zunino L., Olivares F., Scholkmann F., Rosso O.A. (2017). Permutation entropy based time series analysis: Equalities in the input signal can lead to false conclusions. Phys. Lett. A.

[44] Laut I., Räth C. (2016). Surrogate-assisted network analysis of nonlinear time series. Chaos Interdiscip. J. Nonlinear Sci..

[45] Theiler J., Eubank S., Longtin A., Galdrikian B., Doyne Farmer J. (1992). Testing for nonlinearity in time series: The method of surrogate data. Phys. D Nonlinear Phenom..

[46] Schreiber T., Schmitz A. (1996). Improved surrogate data for nonlinearity tests. Phys. Rev. Lett..

[47] Lancaster G., Iatsenko D., Pidde A., Ticcinelli V., Stefanovska A. (2018). Surrogate data for hypothesis testing of physical systems. Phys. Rep..

[48] Goldberger A.L., Amaral L.A.N., Glass L., Hausdorff J.M., Ivanov P.C., Mark R.G., Mietus J.E., Moody G.B., Peng C.K., Stanley H.E. (2000). PhysioBank, PhysioToolkit, and PhysioNet: Components of a new research resource for complex physiologic signals. Circulation.

[49] Schalk G., McFarland D., Hinterberger T., Birbaumer N., Wolpaw J. (2004). BCI2000: A general-purpose brain–computer interface (BCI) system. IEEE Trans. Biomed. Eng..

[50] Capilla A., Arana L., García-Huéscar M., Melcón M., Gross J., Campo P. (2022). The natural frequencies of the resting human brain: An MEG-based atlas. NeuroImage.

[51] Libedinsky C., Livingstone M. (2011). Role of Prefrontal Cortex in Conscious Visual Perception. J. Neurosci..

[52] Bandt C. (2019). Small Order Patterns in Big Time Series: A Practical Guide. Entropy.

[53] Huang M., Sun Z., Donner R.V., Zhang J., Guan S., Zou Y. (2021). Characterizing dynamical transitions by statistical complexity measures based on ordinal pattern transition networks. Chaos Interdiscip. J. Nonlinear Sci..

[54] Zanin M. (2021). Assessing time series irreversibility through micro-scale trends. Chaos Interdiscip. J. Nonlinear Sci..

[55] Gancio J., Masoller C., Tirabassi G. (2024). Permutation entropy analysis of EEG signals for distinguishing eyes-open and eyes-closed brain states: Comparison of different approaches. Chaos Interdiscip. J. Nonlinear Sci..

[56] Räth C., Gliozzi M., Papadakis I.E., Brinkmann W. (2012). Revisiting Algorithms for Generating Surrogate Time Series. Phys. Rev. Lett..

